# *Korrika*, running in collective effervescence through the Basque Country: A model of collective processes and their positive psychological effects

**DOI:** 10.3389/fpsyg.2023.1095763

**Published:** 2023-02-10

**Authors:** Jon Zabala, Susana Conejero, Aitziber Pascual, Larraitz N. Zumeta, José J. Pizarro, Itziar Alonso-Arbiol

**Affiliations:** ^1^Department of Basic Psychological Processes and Development, University of the Basque Country, Donostia-San Sebastián, Spain; ^2^Department of Social Psychology, University of the Basque Country, Donostia-San Sebastián, Spain; ^3^Escuela de Psicología, Universidad Católica del Norte, Antofagasta, Chile; ^4^Department of Clinical and Health Psychology and Research Methods, University of the Basque Country, Donostia-San Sebastián, Spain

**Keywords:** Durkheim, collective gatherings, perceived emotional synchrony, self-transcendent emotions, kama muta, collective empowerment, well-being, belongingness

## Abstract

The neo-Durkheimnian model suggests that feedback and emotional communion between participants during a collective gathering (i.e., perceived emotional synchrony: PES) is one of the key mechanisms of collective processes. This shared emotional experience gives rise, in turn, to more intense emotions, this being one of the explanatory models of the positive psychological effects of collective participation. Through a quasi-longitudinal design of three measurement-times (*N* = 273, 65.9% women; age: 18–70, *M* = 39.43, *SD* = 11.64), the most massive social mobilization that is celebrated in favor of the Basque language in the Basque Country (*Korrika*) was analyzed. Repeated measures and sequential mediation analyzes supported the model. The effect of participation on social integration was mediated by the increase in emotions of enjoyment through PES; the effect on social acceptance, social contribution, and social actualization was mediated by increased kama muta through PES; the effect on collective empowerment was mediated by the increase in self-transcendent emotions through PES; and the effect on remembered well-being was partially mediated by PES. Finally, it was also verified for the first time that the effect of participation on social integration, social acceptance and social actualization was maintained through PES (but not through emotions) for at least 6–7 weeks after the event ended. Also, it is concluded that Kama muta is a relevant emotion during collective gatherings.

## 1. Introduction

The social isolation measures taken in different countries around the world to deal with the pandemic have been associated with higher rates of stress, anxiety, and depression (Marroquín et al., 2020; Bueno-Notivol et al., 2021). Fortunately, these measures have been lifted in most countries of the world, allowing the population to return to social interactions or collective gatherings — social situations in which two or more people meet in one place with a common goal (McPhail and Wohlstein, 1983)—which have been shown to be important for people’s psychological health (Dimmock et al., 2021; Macdonald and Hülür, 2021). Although it has been found that collective participation has a wide variety of positive psychological effects, more efforts have to be made to explain how these effects are generated during collective gatherings.

Through a quasi-longitudinal design, this work aims to analyze the positive psychological effects of participation in *Korrika*, the most important and massive event held in the Basque Country in favor of *euskera* (Basque language). Based on the neo-Durkheimnian model of collective processes proposed by Páez et al. (2015) we will analyze the mechanisms involved in the development of these effects, as well as their durability over time.

### 1.1. Collective gatherings and social movements

Massive collective gatherings contain a high emotional and symbolic charge, and can profoundly mark the individual and collective life of people (Collins, 2004; Durkheim, 1912/1915; Wlodarczyk et al., 2020). A series of studies, in line with Durkheim’s theory (1912/1915), have shown that participation in collective gatherings has a wide variety of positive psychological effects. For example, it has been found that participation in collective gatherings is related to an increase in social cohesion —e.g., integration and social identity, perceived social support and solidarity—, leading to positive effects on social beliefs, such as the benevolence of people and society, and to an increase in empowerment at the individual level —e.g., self-esteem and life satisfaction— and, at the collective level, to a higher collective self-esteem and perceived collective efficacy (Drury and Reicher, 2009; Páez et al., 2011, 2013, 2015; Tewari et al., 2012; Khan et al., 2016; Zumeta L. et al., 2016; Wlodarczyk et al., 2017b; Bouchat et al., 2020).

Among these psychological effects, collective identity has been highlighted as one of the most important factors that predict collective action, which in turn predicts collective efficacy, another of the most important factors of social mobilizations (van Zomeren et al., 2008; Agostini and van Zomeren, 2021). Therefore, collective gatherings could be considered important fuels for social mobilizations (Pizarro et al., 2022).

Finally, collective gatherings have also been shown to be important resources to counteract the negative effects of painful and traumatic events such as attacks, natural disasters, or social isolation during pandemic. In the face of these events, the strengthening of solidarity, support, and social cohesion during collective gatherings helps in the post-traumatic process (Páez et al., 2007; Wlodarczyk et al., 2016, 2017a; Pelletier, 2018; Zlobina and Dávila, 2022).

However, it is still necessary to clarify the psychological mechanisms involved in producing these effects. Because mere participation in collective gatherings does not necessarily provoke particular effects (see Thonhauser, 2022; see also Collins, 2004), outcomes generated in these instances of social life should involve the activation of one or more mechanisms.

### 1.2. Collective effervescence

Durkheim’s theoretical model (Durkheim, 1912/1915) on collective processes provides a series of key elements to answer this question. Durkheim, who was interested in knowing what makes people stick together in society, developed the theory of collective effervescence. He maintained that collective gatherings are fundamental pillars for the individual and collective life of people. These gatherings, periodically held, fulfill the role of recreating the social group and reviving shared values. Participation in such gatherings fosters social cohesion and leaves participants with a renewed confidence in society and a sense of energy on an individual and collective level.

For these positive psychological effects to take place, collective effervescence is necessary. Durkheim described collective effervescence as a collective emotional exaltation that arises from contagion and emotional feedback between the people gathered. Each emotion resonates and feeds back among the participants, reaching a point of exaltation and emotional communion. Regardless of the type of affect that predominates during the collective gathering —for example, sadness at a funeral, joy at a celebration or anger at a demonstration—, the positive psychological effects of collective participation are the result of emotional communion, that is, from the shared emotional experience and the feelings of unity derived from it (Páez et al., 2015).

### 1.3. Perceived emotional synchrony

Páez et al. (2015), intending to empirically contrast Durkheim’s approaches, proposed the Perceived Emotional Synchrony (PES) as a measure of collective effervescence; it is described “as an emotional experience felt by participants during group gatherings, involving a sense of togetherness” (Wlodarczyk et al., 2021, p. 3). Various meta-analyses have shown that experimentally induced synchronous behaviors foster perceived social bonding and positive affect (e.g., Rennung and Göritz, 2016; Mogan et al., 2017). Synchronous behaviors such as singing, dancing, and repeating the same gestures and shouts are common during gatherings. These synchronous and symbolic behaviors during collective meetings awaken an emotional energy that intensifies and feeds back among the participants, giving rise to a shared emotional experience, reciprocal empathy and collective consciousness, that is, collective effervescence or PES (Durkheim, 1912/1915; Collins, 2004; Páez et al., 2015; Wlodarczyk et al., 2020, 2021).

Through this neo-Durkheimian model proposed by Páez et al. (2015) and subsequent studies that have followed this line of research, it has been found that the psychological effects of collective gatherings do not take place by mere collective participation, but rather that these effects are facilitated by the PES (Páez et al., 2015; Zumeta L. N. et al., 2016; Pelletier, 2018; Pizarro et al., 2019; Bouchat et al., 2020; Wlodarczyk et al., 2020; Zumeta et al., 2020; Castro-Abril et al., 2021; Wlodarczyk et al., 2021; Pizarro et al., 2022). These studies suggest that the PES is one of the most important mechanisms of collective processes. The emotional communion generated from the emotional feedback between the participants gives rise, in turn, to more intense emotions during the collective gathering. As a result of this process, the participants return to their daily lives revitalized, that is, with a greater sense of social cohesion or integration, trust and empowerment at the individual and collective level (Páez et al., 2015; Wlodarczyk et al., 2020; Zumeta et al., 2020, 2021; Wlodarczyk et al., 2021; Pizarro et al., 2022).

In the quasi-longitudinal study by Wlodarczyk et al. (2021), the relationships between PES, enjoyment emotions and self-transcendent emotions and their effects in a folk ritual were analyzed through structural equation models. PES acted as a direct predictor of increased self-transcendent emotions and emotions of enjoyment, as well as increased social integration, beliefs of a benevolent world and collective self-esteem. However, indirect effects of PES were also found through self-transcendent emotions on benevolent world beliefs and ingroup solidarity, as well as through enjoyment emotions on social integration.

These results show that the self-transcendent emotions and the emotions of enjoyment fostered by the PES during a gathering can also facilitate the effects of collective participation (i.e., the effects that the participants carry with them after leaving the collective situation). Based on this process, and on the existing literature on the effects of collective effervescence, we may refer to *proximal and distal effects* of PES (Wlodarczyk et al., 2020; Zumeta et al., 2020; Pizarro et al., 2022).

#### 1.3.1. Positive emotions and kama muta during collective effervescence

The most immediate or proximal effects of PES are those that occur during the same effervescence or collective situation, for example, positive self-transcendent emotions (Pizarro et al., 2022). Self-transcendent emotions (e.g., hope, inspiration, and feeling grateful and amazed) bring a person out of their reverie by making them more receptive to stimuli from the social and natural world around them (Haidt, 2006; Fredrickson, 2009; Van Cappellen and Rimé, 2014). These emotional states induce an experience of self-transcendence, that is, a mental state in which the salience of the self decreases or in which feelings of connection with other people or entities increase (Yaden et al., 2017). This transcendence or connection induced by self-transcendent emotions facilitates, for example, the acceptance of people and the society they make up, as well as interest in their well-being (Aquino et al., 2011; Stellar et al., 2017; Pizarro et al., 2021). Likewise, specifically in the case of the feeling of being moved and hope, these are significant factors related to collective efficacy and the action implied in social movements (Wlodarczyk et al., 2017b; Cohen-Chen and Van Zomeren, 2018; Landmann and Rohmann, 2020; Zumeta et al., 2021).

Another possible proximal effect of the PES, and to which a self-transcendental character can be attributed, is *kama muta*. Kama muta is an emotion used to refer to being moved by love of others (Fiske et al., 2017; Zickfeld et al., 2019). It is based on the horizontal sharing of relationships and arises from the sudden activation of these relationships in which people perceive each other as an equal in some essential aspect that they share (Fiske, 1992; Fiske et al., 2017; Seibt et al., 2019). For example, reunions, reconciliations and acts of friendship are some of the experiences that can evoke kama muta (Alfaro-Beracoechea and Contreras-Tinoco, 2021). During the emotional experience of kama muta, people feel mutual love, identification, solidarity, pity, kindness and devotion, promoting the desire to commit more strongly in those relationships (Fiske et al., 2017; Zickfeld et al., 2019).

We currently have no evidence of previous studies that have analyzed the role of kama muta during collective gatherings; however, it has been shown that one of the evocative experiences of kama muta are the big collective gatherings (Alfaro-Beracoechea and Contreras-Tinoco, 2021). In addition, some works suggest that social movements can arouse kama muta and induce a sense of moral commitment toward other people, for example, arousing the motivation to support causes related to a group (Fiske et al., 2017; Seibt et al., 2019; Landmann and Rohmann, 2020; Lizarazo Pereira et al., 2022). We believe that PES can be a strong trigger for kama muta, especially during a collective gathering in which people come together for a common cause, as in the case of this study.

Lastly, the emotions of enjoyment, such as joy and fun, are expressed collectively during collective gatherings; although they cannot be considered self-transcendent emotions, in some studies they have also been related to feelings of unity and increased social integration (Novelli et al., 2013; Wlodarczyk et al., 2021).

#### 1.3.2. Well-being and collective empowerment after collective effervescence

Distal effects are the effects that last or extend beyond the collective effervescence (i.e., the effects that the participants take with them into their daily lives after leaving the gathering). As mentioned above, people’s evaluation of social cohesion, social beliefs and empowerment usually improve after collective gatherings (Pizarro et al., 2022). These aspects are often closely related to people’s psychological health, for example, to *social well-being*.

Social well-being is the evaluation that people make of the circumstances and functioning they have within society (Keyes, 1998), and is made up of five dimensions: social integration, social acceptance, social contribution, social actualization and social coherence. *Social integration* refers to the feeling of belonging and being accepted by other people who constitute their social reality and the degree of similarity perceived with them. Social belonging is a good indicator of people’s psychological health, and a factor that protects against anxiety, stress and depression (Postmes et al., 2019; Zabala et al., 2020; Páez and Oyanedel, 2021). *Social acceptance* refers to the positive view of human nature and trust in others. According to Keyes (1998), this belief about people’s benevolence is also an important indicator of people’s health, since psychologically healthier people tend to attribute their own benevolence and personal acceptance —acceptance of bad things and good— also to other people. This trust placed in other people is accompanied by *social contribution*: the belief that oneself is also a useful member of society. This dimension is based on perceived self-efficacy and sense of control (Bandura, 1997; Keyes, 1998), relevant factors in people’s psychological health. As for *social actualization*, this refers to the belief that society progresses in a beneficial direction for people. Lastly, the psychologically healthier people, in addition to placing trust in oneself, in the people and the society they make up, also tend to worry about knowing and understanding the social life that surrounds them; this is what Keyes (1998) named *social coherence*, the feeling that one is able to understand what is happening around him.

After collective gatherings, people’s evaluation of quality of life tends to improve even in such personal aspects as self-esteem, sense of control and autonomy, as well as meaning and satisfaction with life (Páez et al., 2015; Zumeta L. et al., 2016; Wlodarczyk et al., 2021), aspects included in the *remembered well-being* of Hervás and Vázquez (2013).

Finally, collective gatherings in addition to being beneficial at the individual level, can also be beneficial at the collective level, and increase perceived collective efficacy —the shared belief of a group in its ability to organize and execute the actions required to reach certain levels of achievement (Bandura, 1997; Zumeta L. et al., 2016; Zumeta et al., 2020, 2021). When it comes to minority or discriminated groups, and this perceived efficacy is aimed at counteracting existing power relations and influencing social change, it is referred to as *collective psychological empowerment* (Drury and Reicher, 2009), which may also be strongly related to the well-being of people (Zabala et al., 2020).

### 1.4. The durability of the effect of participation

Durkheim stresses the importance of the regular practice of collective gatherings since the effect of participation is diluted over time. The durability of the effect of collective participation has been analyzed on very few occasions. In the study by Pizarro et al. (2019) the effect of participation in a mindful-dancing program dissipated within 1 week of participation. In contrast, in other studies, the effect of participation was maintained for at least 3 weeks (Páez et al., 2007, 2015; Rimé et al., 2010) and 4 weeks after participation (Páez et al., 2011; Tewari et al., 2012; Khan et al., 2016). In the study by Bouchat et al. (2020) the distal effects of the PES during the most massive event of scouts were analyzed. PES during the encounter predicted scores for some of the distal effects after 10 weeks of participation. In the present study, we will analyze the effects of participation up to 6–7 weeks after having participated.

### 1.5. Current study: Korrika, the claim of the language and the culture on the territory

Korrika^1^ is a crowded race of a leisure nature that every 2 years crosses the Basque Country (a region currently split between Spain and France) for 11 days without interruption. The objective of this ritual is to raise funds to promote Euskera, a minority^2^ and isolated language spoken in the Basque Country, whose origin is a mystery and that continues to challenge many scholars to decipher it. Basque is an essential element, almost sacralized for the identity of Basque speakers, and is estimated that around six hundred thousand people participate in Korrika, making this ritual a highly emotional and indescribable celebration for many participants (del Valle, 1988).

Korrika contains all the elements of a collective ritual and therefore the conditions for the collective effervescence to arise (i.e., PES), as well as the emotions of enjoyment, self-transcendent emotions and kama muta. The ritual, as a whole, is made up of symbolic behaviors that represent and claim the territory of the Basque language and the entire Basque culture. Each group of participants travels a section of the route while carrying a baton with a secret message inside that is revealed in the massive celebration at the end of the route. This act of going through the entire territory with a baton that passes from hand to hand symbolizes the legacy of Basque —that Basque does not stop, does not disappear as long as there are runners (i.e., speakers) who practice it—. Both at the beginning and at the end, as well as throughout the race, repetitive, stereotyped and synchronous behaviors are expressed, such as clapping, singing and shouting. This is accompanied by music and symbolic elements that express shared values, such as flags and traditional elements in clothing (del Valle, 1988). This great social mobilization around the Basque language is likely to be explained by the strong identity and collective empowerment of the Basque society. However, we believe that between these factors and the collective effervescence there is mutual feedback that strengthens the social movement, and that is also crucial.

### 1.6. Objectives and hypotheses

The present study has three objectives:

(1) To test whether participation in the Korrika collective ritual fosters social well-being, remembered well-being and collective empowerment (dependent variables).
(2) To examine if the effect (if any) of the quality of participation on the dependent variables is mediated by the PES and its proximal effects (emotions of enjoyment, self-transcendent emotions and kama muta) in this sequence, and after controlling the effect of the dependent variables before having participated in Korrika.
(3) To analyze the durability of the effect of participation up to 6–7 weeks after having participated, and analyze the effect of the same mediators variables (PES and proximal effects) on that dependent variables where the effect of the participation still endures.

Related to these objectives, the following hypotheses were formulated:

*H1*: Participants are expected to express higher social well-being, remembered well-being and collective empowerment after having participated in Korrika.

*H2*: The effect of the quality of participation on the dependent variables is expected to be mediated by the PES, or by the increase in the emotions of enjoyment, self-transcendent emotions and kama muta through the PES.

*H3*: The durability of the effect of participation up to six-seven weeks after Korrika has finished will be explored. No assumptions are made in this regard. However, if the effect of participation persists on some dependent variables, it is expected that the PES or its proximal effects will predict positively that effect.

## 2. Materials and methods

### 2.1. Participants

Three questionnaires were administered in three times. The number of people who answered the questionnaire of Time 1 was 748 (63.1% women and 36.9% men), aged between 18 and 73 years (*M* = 39.28; *SD* = 12.13). From that sample only 404 people (66.1% women and 32.2% men), aged between 18 and 73 years (*M* = 39.79, *SD* = 11.74) answered also the questionnaire of Time 2. Finally, from that sample (T1-T2), 273 people answered the questionnaire of Time 3 (65.9% women and 33.01% men), aged between 18 and 70 years (*M* = 39.43, *SD* = 11.64). All the participants were citizens from the historical territory of the Basque Country.

### 2.2. Procedure

Given that our objective in this study was to test hypotheses of causal relationships, and despite the limitations posed by quasi-experimental studies, and the reality that we intended to study, we believed that it was the most appropriate design to achieve our objectives. Data was collected in three times. The first survey was administered 3 weeks before starting Korrika (Time 1: T1). The second survey was administered between the first and seventh days after participating in Korrika (Time 2: T2). Finally, the third survey was administered between 6 and 7 weeks after Korrika had finished (Time 3: T3). The data of the dependent variables —i.e., measures of the distal effects— (social well-being, remembered well-being and collective empowerment) were collected at T1, T2, and T3. Data on quality of participation, PES, enjoyment emotions, self-transcendent emotions, and kama muta were collected at T2.

Once the approval of the Ethics Committee of the university to which the authors belong was obtained (M10_2019_004), there were collected the emails of those people who intended to participate in Korrika 2019 and in the research. *Alfabetatze eta Euskalduntze Koordinakundea* (AEK; the association that organizes the Korrika event) collaborated with the research team by sending an email invitation to all Korrika collaborators, requesting their voluntary participation in the research. On the other hand, researchers went to the capitals of the Basque Country and collected the emails from those who had been willing to participate in Korrika and in this research. Participants answered all questionnaires online through the *Qualtrix XM* platform.

### 2.3. Instruments

*Quality of participation* (Páez et al., 2015). The involvement of the participants in Korrika was measured with three items on a 7-point scale (1 = *Not at all*, 7 = *Very much*). For example: “How intense was your participation? “. Obtained reliability indexes were satisfactory (*α* = 0.83; *Ω* = 0.84).

*Perceived Emotional Synchrony* (PES; Páez et al., 2015, brief version by Wlodarczyk et al., 2020). Through four items, the extent to which the participants experienced collective effervescence was analyzed on a 7-point scale (1 = *Not at all*, 7 = *A lot*). For example: “We felt a strong shared emotion.” Obtained reliability indexes were satisfactory (*α* = 0.92; *Ω* = 0.92).

*Enjoyment and Self-Transcendent Emotions* (Modified Differential Emotions Scale – MDES; Fredrickson, 2009). The extent to which participants felt two enjoyable emotions (amusement/entertainment and joy/happiness) and four self-transcendent positive emotions (wondered, grateful, inspired, and hopeful) was analyzed on a 5-point ordinal scale (1 = *Not at all*, 2 = *A little bit*, 3 = *Moderately*, 4 = *Quite a bite* y 5 = *Extremely*). For example: “What is the most joyful, glad, or happy you felt?” or “What is the most grateful, appreciative, or thankful you felt?.” Obtained reliability indexes were satisfactory: *r* = 0.53 (*α* = 0.68) and *α* = 0.78 (*Ω* = 0.78) respectively.

*Kama muta* (KAMUS; Zickfeld et al., 2019). The extent to which participants had experienced kama muta was analyzed on a 7-point scale (1 = *Not at all*, 7 = *Very much*). The data of the evaluation dimensions, of four items, were analyzed; for example: “An exceptional sense of closeness appear” and of motivation (also made up of four items); for example: “I wanted to hug someone.” Obtained reliability indexes were satisfactory, *α* = 0.92 (*Ω* = 0.93).

*Social Well-Being* (SWB; Keyes, 1998, adapted by Blanco and Díaz, 2005). Using a 5-point scale (1 = *Strongly disagree*, 5 = *Strongly agree*), the following dimensions were analyzed: social integration (e.g., “I feel close to other people in my community”), social acceptance (e.g., “I think that people are basically good”), social contribution (e.g., “I have something valuable to give the world”) and social actualization (e.g., “Society is making progress, getting better”). Each dimension was made up of three items, as in the original version of 15 items (Keyes, 2002). We did not analyze the social coherence dimension. The reliability indices in social integration ranged from *α* = 0.69 (*Ω* = 0.70) at T1 and *α* = 0.84 (*Ω* = 0.85) at T3. In social contribution range was between *α* = 0.75 (*Ω* = 0.76) and *α* = 0.81 (*Ω* = 0.82), and in social actualization between *α* = 0.83 (*Ω* = 0.83) and *α* = 0.85 (*Ω* = 0.86). For social acceptance, reliability indices were lower: between *α* = 0.48 (*Ω* = 0.50) at T1 and *α* = 0.62 (*Ω* = 0.64) at T2.

*Remembered Well-Being* (The Pemperton Happiness Index – PHI; Hervás and Vázquez, 2013). Through 10 items, the participants’ recalled well-being was analyzed on a 10-point scale (1 = *Strongly disagree*, 10 = *Strongly agree*). Data on general well-being (e.g., “I am very satisfied with my life”), eudaimonic (e.g., “I think my life is useful and worthwhile”) and hedonic (e.g., “I enjoy a lot of little things every day”) was collected. However, the hedonic dimension was removed from the analysis, because the only inverse item (i.e., “I have a lot of bad moments in my daily life”) showed a considerably small factor loading, and this negatively affected the fit indices. Indeed, we found that a lot of participants responded to this item in the opposite direction in which the answer should have been given. Both dimensions were treated as an only measure of well-being. Obtained reliability indexes were satisfactory, ranging between *α* = 0.90 (*Ω* = 0.90) in T1 and *α* = 0.93 (*Ω* = 0.93) in T3.

*Collective Empowerment* (Collective Efficacy Questionnaire for Sports – CEQS; Martínez et al., 2011). The group efficacy (that is, as Basque speakers) perceived by the participants was measured with four items on a 10-point scale (1 = *Not at all capable*, 10 = *Very capable*). For example, “To carry out actions.” In addition, we added two items elaborated *ad hoc* to capture Drury and Reicher’s (2009) concept of collective psychological empowerment, specifically: “To promote social change” and “To achieve common goals.” Obtained reliability indexes were satisfactory, ranging between *α* = 0.89 (*Ω* = 0.89) in T1 and *α* = 0.91 (*Ω* = 0.91) in T3.

### 2.4. Data analysis

We conducted ANOVA repeated measures analysis in order to test Objectives 1 and 3. To analyze the effects of the mechanisms involved in changes from T1 to T2 (i.e., Objective 2), we conducted several structural equation models using the quality of participation as the main predictor, and a sequential mediation approach from PES to the emotional aspects (i.e., emotions of enjoyment, self-transcendent emotions and kama muta). Finally, for each dependent variable we included the score in T1 to calculate specifically possible changes due to having participated in the ritual. Finally, to analyze the durability of the effect of the mechanisms until T3 (i.e., Objective 3), we first performed linear regressions on the dependent variables where the effect of participation persisted, to then carry out a series of structural equation models that were most consistent with the previous results and the fit indicators. For these models, no outliers were excluded, and the proposed theoretical model was compared with other alternative models, which was shown to be more adequate both theoretically and statistically (See Supplementary Tables S2, S3).

To evaluate the fit of the models, we included the chi-square test^3^ together with CFI (Comparative Fit Index) and TLI (Tucker–Lewis Index), whose values over 0.90 are considered acceptable. Likewise, RMSEA (Root Mean Square Error of Approximation) and SRMR (Standardized Root Mean Square Residual) were taken into account, whose values below 0.05 indicate a good model, and with values between 0.05 and 0.08 indicating that the model is reasonably good (Hu and Bentler, 1999). The applied estimation procedure was *Maximum Likelihood* and all analyses were conducted through JASP 0.15.

Concerning the participants, Objective 1 and Objective 2 were accomplished with the sample of participants who responded to T1 and T2. Instead, Objective 3 was accomplished with the sample of participants who had answered the questionnaires in T1, T2, and T3. There were no statistically significant differences between these groups neither in gender, nor in age, nor in the dependent variables before having participated in Korrika (see Supplementary Table S1).

## 3. Results

### 3.1. Descriptive and preliminary analyses

Means and standard deviations, correlations, and collinearity statistics are shown in Supplementary Tables S2–S4. All the analyzed variables in T2 were related significantly and positively. Correlation ranged from *r* = 0.16 between the quality of participation and social actualization to *r* = 0.70 between enjoyment and self-transcendent emotions; all *p*s < 0.001 (see Supplementary Table S3).^4^

### 3.2. Effect of participation on dependent variables

The results of the repeated measures ANOVAs (see Table 1) show that the scores of the dependent variables in T1 increase significantly in T2, except in the case of remembered well-being. Furthermore, it is worth mentioning that the other effect sizes —except for collective empowerment— are satisfactory, especially in the case of social actualization. In general, we observed an increase in the scores of the dependent variables from T1 to T2; therefore, Hypothesis 1 was confirmed.

**Table 1:** Results of repeated measures of dependent variables in T1 and T2.

Dependent variables	T1	T2			
	*M* (SD)	*M* (SD)	*F*(1, 403)	*p*	$\eta_{p}^{2}$
Social integration	3.98 (0.60)	4.15 (0.64)	31.626	0.001	0.07
Social acceptance	3.20 (0.60)	3.41 (0.67)	37.390	0.001	0.09
Social contribution	3.60 (0.67)	3.74 (0.73)	19.377	0.001	0.05
Social actualization	2.52 (0.77)	2.94 (0.83)	134.870	0.001	0.25
Remembered well-being	7.89 (1.10)	7.97 (1.17)	3.303	0.070	0.01
Collective empowerment	7.78 (1.27)	8.02 (1.34)	14.143	0.001	0.03

### 3.3. Sequential mediation analysis

**Figure 1:**
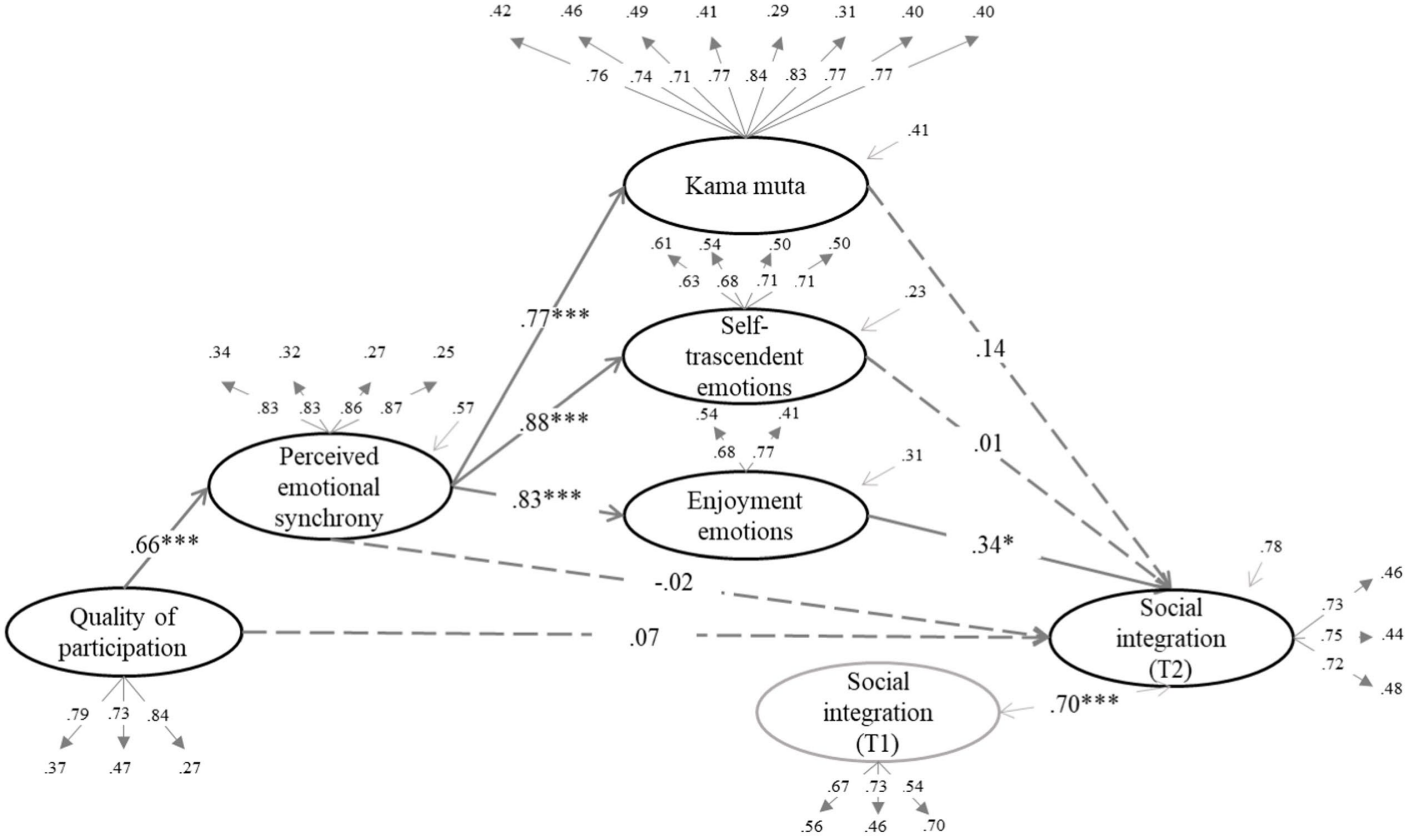
Perceived emotional synchrony and emotions as mediators of the effect of quality of participation on social integration controlling for pre-participation scores. Model fit: χ^2^ = (404, 313) = 811.975, *p* < 0.001, CFI = 0.920, TLI = 0.910, RMSEA = 0.063, SRMR = 0.058.

**Figure 2:**
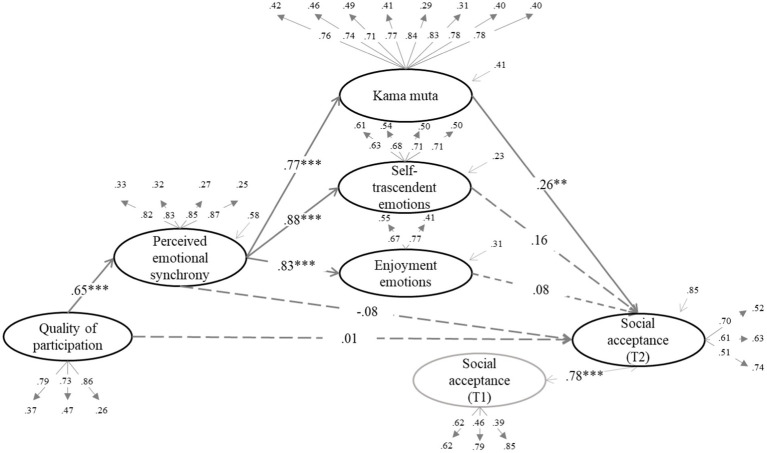
Perceived emotional synchrony and emotions as mediators of the effect of quality of participation on social acceptance controlling for pre-participation scores. Model fit: χ^2^ = (404, 313) = 904.186, *p* < 0.001, CFI = 0.900, TLI = 0.888, RMSEA = 0.068, SRMR = 0.060.

**Figure 3:**
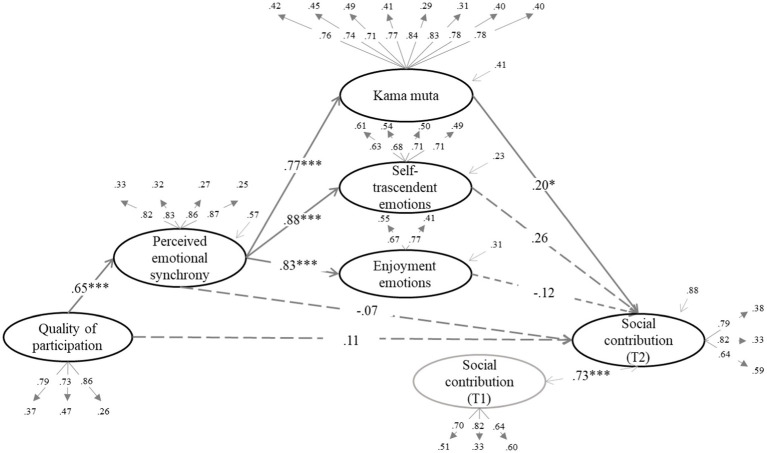
Perceived emotional synchrony and emotions as mediators of the effect of quality of participation on social contribution controlling for pre-participation scores. Model fit: χ^2^ = (404, 313) = 881.122, *p* < 0.001, CFI = 0.912, TLI = 0.901, RMSEA = 0.067. SRMR = 0.074.

**Figure 4:**
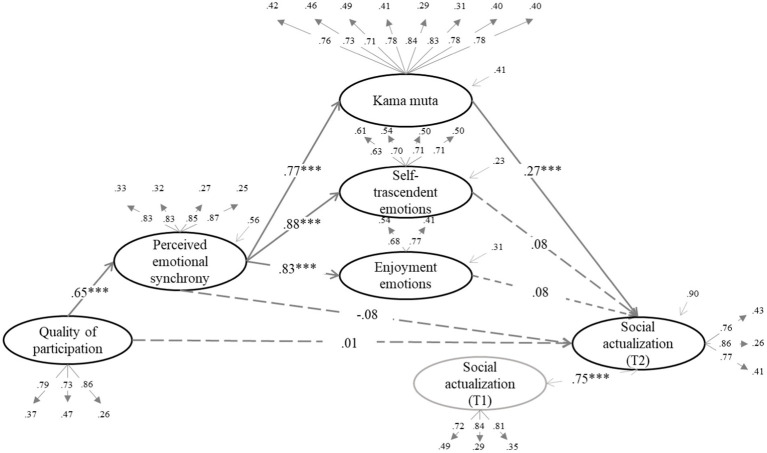
Perceived emotional synchrony and emotions as mediators of the effect of quality of participation on social actualization controlling for pre-participation scores. Model fit: χ^2^ = (404, 313) = 816.599, *p* < 0.001, CFI = 0.925, TLI = 0.916, RMSEA = 0.063. SRMR = 0.056.

**Figure 5:**
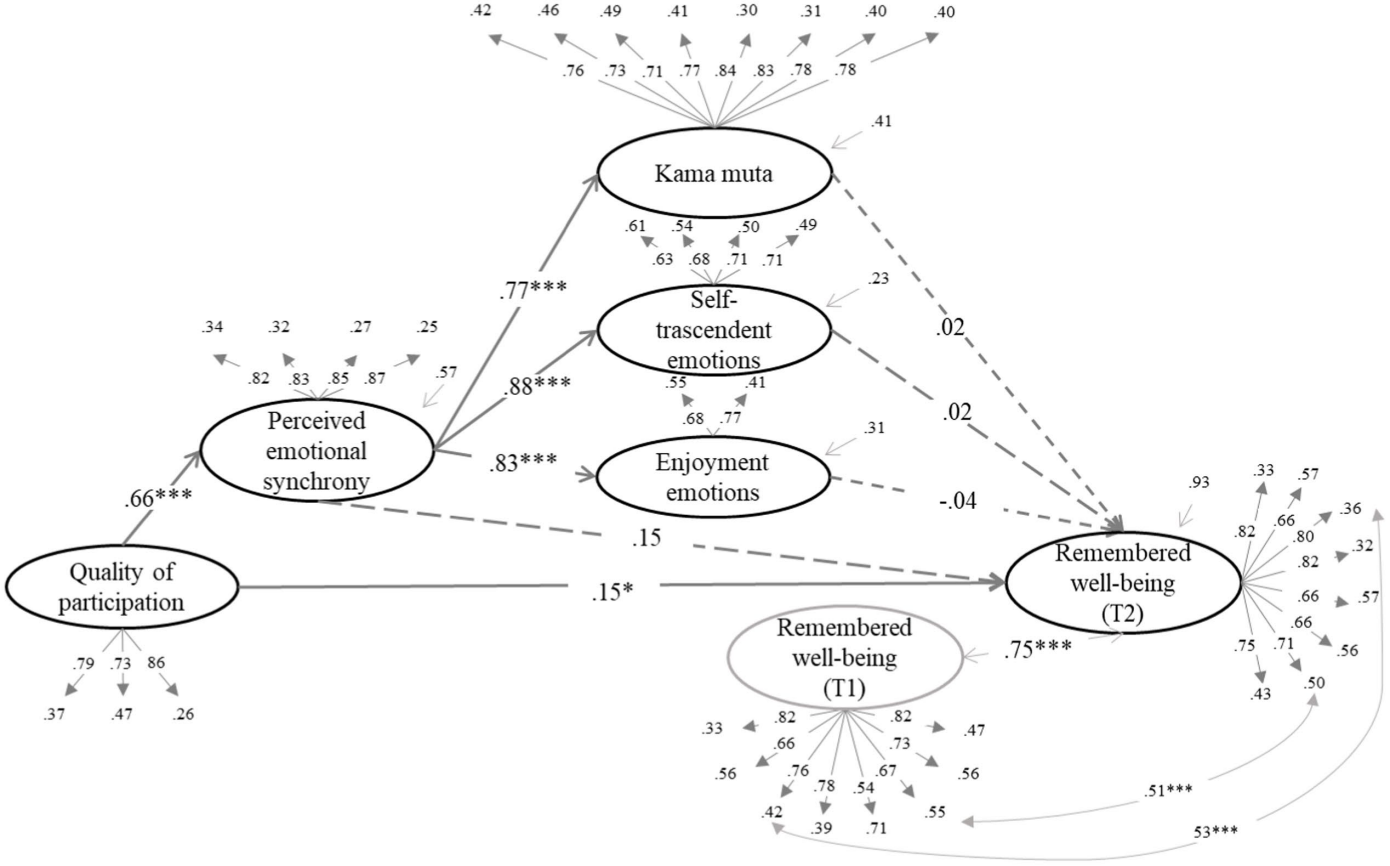
Perceived emotional synchrony and emotions as mediators of the effect of quality of participation on remembered well-being controlling for pre-participation scores. Model fit: χ^2^ = (404, 616) = 1419.787, *p* < 0.001, CFI = 0.914, TLI = 0.907, RMSEA = 0.057, SRMR = 0.056.

**Figure 6:**
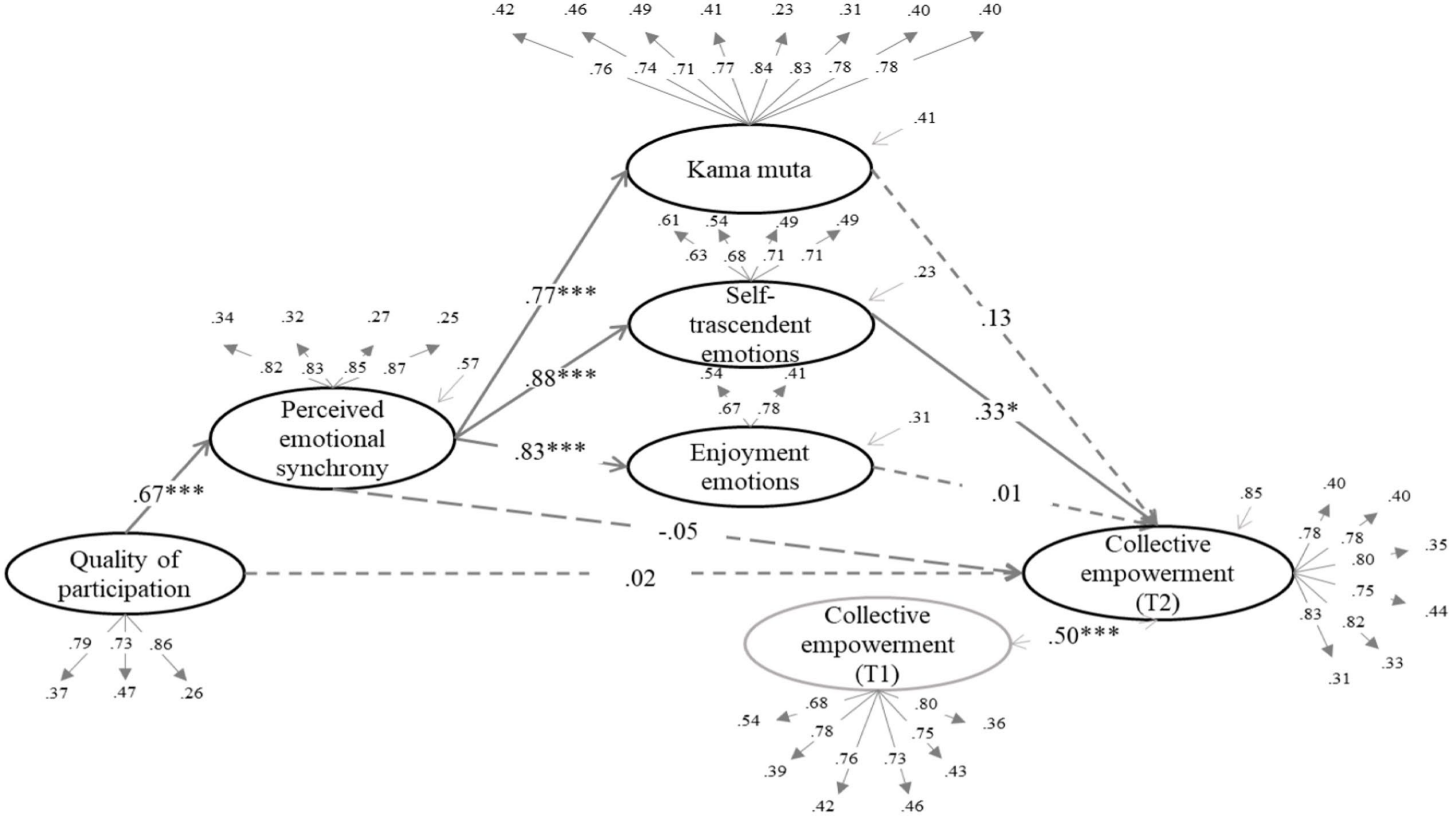
Perceived emotional synchrony and emotions as mediators of the effect of quality of participation on collective empowerment controlling for pre-participation scores. Model fit: *χ*^2^ = (404, 484) = 1237.669, *p* < 0.001, CFI = 0.912, TLI = 0.904, RMSEA = 0.062. SRMR = 0.062.

We tested whether the effect of quality of participation (QP) on the dependent variables was mediated by increased enjoyment emotions, self-transcendent emotions, and kama muta through PES. The structural equation models can be seen in Figures 1–6 (the coefficients shown in the figures are standardized coefficients and the solid arrows indicate that the coefficient is statistically significant). To check the sequential mediation, we calculated the indirect effects of the QP through PES and the proximal effects (emotions of enjoyment, self-transcendent emotions and kama muta) in this sequence (standardized effects and confidence intervals are presented).

#### 3.3.1. Emotions of enjoyment, self-transcendent emotions and kama muta

As it can be seen in all the figures mentioned above, the QP predicts the increase in PES and, in turn, PES acts as a direct predictor of the increase in emotions of enjoyment, of emotions of self-transcendent and kama muta. It is worth mentioning that in another alternative model enjoyment emotions did not predict PES, and that the betas of self-transcendent and kama muta emotions on PES were lower than the betas of PES on them (see Supplementary Table S3). This result further reinforces the proposed model.

#### 3.3.2. Effects on social well-being: Social integration, social acceptance, social contribution, and social actualization

In Figure 1 we can see that there is a direct effect of enjoyment emotions on social integration (*B* = 0.34, *SE* = 0.14, *p* = 0.017, 95% CI [0.060, 0.611]). In the absence of the direct effect of PES on social integration, we analyzed the indirect effect of QP through PES and enjoyment emotions in this sequence. The indirect effect was statistically significant (*B* = 0.19, *SE* = 0.04, *p* = 0.020, 95% CI [0.015, 0.177]); therefore, we could state that the PES mediated the effect of the QP on social integration through of increased emotions of enjoyment.

In Figure 2–4 we can see that there is a direct effect of kama muta on social acceptance (*B* = 0.26, *SE* = 0.04, *p* = 0.008, 95% CI [0.029, 0.190]), social contribution (*B* = 0.20, *SE* = 0.04, *p* = 0.011, 95% CI [0.022, 0.165]) and social actualization (*B* = 0.27, *SE* = 0.04, *p* = 0.001, 95% CI [0.058, 0.202]). Again, in the absence of the direct effect of PES, we calculated the indirect effect of QP through PES and kama muta, which was statistically significant in all three cases: on social acceptance (*B* = 0.13, *SE* = 0.03, *p* = 0.009, 95% CI [0.019, 0.137]), social contribution (*B* = 0.10, *SE* = 0.03, *p* = 0.012, 95% CI [0.014, 0.119]), and social actualization (*B* = 0.13, *SE* = 0.03, *p* = 0.001, 95% CI [0.040, 0.147]). In these cases, PES mediated the effect of QP on the three variables through the increase in kama muta.

#### 3.3.3. Effect on remembered well-being

In Figure 5 we can see that there is a direct effect of the QP on remembered well-being (*B* = 0.15, *SE* = 0.08, *p* = 0.029, 95% CI [0.018, 0.333]), and unlike the previous models, neither the PES neither did proximal effects mediate the effect of QP on remembered well-being. However, in the absence of direct effects of PES and proximal effects, we calculated its total indirect effect, which was statistically significant (*B* = 0.09, *SE* = 0.04, *p* = 0.010, 95% CI [0.026, 0.195]). Alternatively, we also performed a simple mediational analysis [*χ*^2^ = (404, 223) = 603.384, *p* < 0.001, CFI = 0.933, TLI = 0.923, RMSEA = 0.065, SRMR = 0.054], in which PES partially mediated the relationship between QP and remembered well-being (*B* = 0.13, *SE* = 0.06, *p* = 0.007, 95% CI [0.045, 0.286]). Therefore, we can say that PES and proximal effects also influenced the effect of QP on remembered well-being.

#### 3.3.4. Effect on collective empowerment

In Figure 6 we can observe a direct effect of self-transcendent emotions on collective empowerment (*B* = 0.33, *SE* = 0.33, *p* = 0.031, 95% CI [0.065, 1.351]). In turn, we calculated the indirect effect of QP through PES and self-transcendent emotions, which also turned out to be statistically significant (*B* = 0.19, *SE* = 0.12, *p* = 0.033, 95% CI [0.020, 0.476]). In this case, we can say that PES mediated the effect of QP on collective empowerment through self-transcendent emotions.

### 3.4. Durability of effect of participation

**Figure 7:**
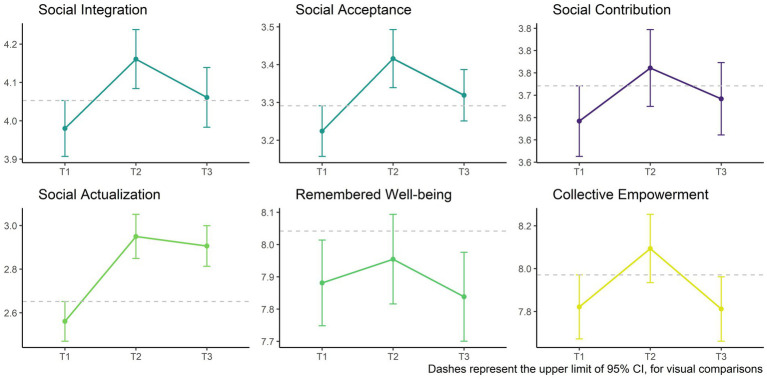
Results of repeated measures of dependent variables in T1, T2, and T3.

As can be seen in Figure 7, scores of social contribution, remembered well-being, and collective empowerment return to their pre-participation status after 6–7 weeks. However, scores for social integration, social acceptance and social actualization remain higher at T3 (i.e., 6–7 weeks after Korrika) than in T1.

In addition, as we can see in Table 2 the unique predictor variable of the scores of social integration, social acceptance and social actualization at T3 is PES, and not the proximal effects. These results were also supported by structural equation modeling (see Supplementary Figures S1–S3), where the PES is the unique predictor of social integration^5^ (*B* = 0.28, *SE* = 0.06, *p* = 0.004, 95% CI [0.059, 0.304]), social acceptance^6^ (*B* = 0.33, *SE* = 0.05, *p* = 0.010, 95% CI [0.031, 0.219]), and social actualization^7^ (*B* = 0.22, *SE* = 0.07, *p* = 0.037, 95% CI [0.009, 0.282]). Enjoyment emotions do not mediate the effect of PES on social integration (*B* = −0.02, *SE* = 0.12, *p* = 0.835, 95% CI [−0.270, 0.218]), and kama muta neither mediates the effect of PES on social acceptance (*B* = −0.07, *SE* = 0.03, *p* = 0.543, 95% CI [−0.080, 0.042]), nor on social actualization (*B* = −0.12, *SE* = 0.05, *p* = 0.214, 95% CI [−0.148, 0.033]).

**Table 2:** Multiple linear regressions of the effect of the quality of participation, of perceived emotional synchrony and of proximal effects on social integration, social acceptance and social actualization on T3, controlling for T1 scores.

	S. Integration^T3^		S. Acceptance^T3^		S. Actualization^T3^
	*B*		*B*		*B*
S. Integration^T1^	0.51***	S. Acceptance^T1^	0.43***	S. Actualization^T1^	0.58***
Quality of participation	−0.08	Quality of participation	−0.12	Quality of participation	−0.06
PES	0.13*	PES	0.21**	PES	0.16*
Enjoyment emotions	0.03	Kama muta	0.02	Kama muta	−0.10
*R* ^2^	0.31		0.24		0.35
*F*(4, 268)	29.357***		20.753***		36.689***

B, standardized beta; *p < 0.05; **p < 0.01; ***p < 0.001.

In summary, the changes in the dependent variables from T1 to T2 are positive and statistically significant except for remembered well-being. All QP effects were mediated by some proximal effect of PES, except for remembered well-being. However, even in this case, apart from being a total indirect effect of PES and proximal effects, in simple mediational analyses PES partially mediated the effect of QP; therefore, in these cases Hypothesis 2 was partially confirmed. Finally, the effect of PES —but not of the proximal effects— was maintained on social integration, social acceptance and social actualization until T3 (i.e., at least up to 6–7 weeks after participation), also confirming Hypothesis 3.

## 4. Discussion

The Korrika collective ritual offers an excellent setting to investigate collective processes. This ritual symbolizes the vindication and legacy of the Basque language and culture over the historical territory and it contains a great emotional and symbolic charge, as well as all the essential elements for the collective effervescence to arise. The results of this semi-longitudinal and quasi-experimental study support the neo-Durkheimnian model of collective processes proposed by Páez et al. (2015).

On the one hand, it has been found that participation in Korrika effectively fosters social cohesion, renews trust in society and empowers individuals and groups. Specifically, we have found an increase in social integration after participation in Korrika, as well as positive effects on acceptance and social actualization. In turn, empowerment at the individual level, that is, social contribution, and empowerment at the collective level have also been favored. The trend increase in remembered well-being may be due to the fact that T1 scores were already high before participating in Korrika. In any case, previous studies show statistically significant effects on remembered well-being (Páez et al., 2015; Zumeta L. et al., 2016; Wlodarczyk et al., 2021).

On the other hand, in line with the neo-Durkheimian model, the results show that PES and its proximal effects (Páez et al., 2015; Wlodarczyk et al., 2020, 2021) facilitate these positive psychological effects. First, QP predicted PES, and PES was shown to be a predictor of enjoyment emotions, self-transcendent emotions, and kama muta. Thus, the proximal effects of PES facilitated all the effects of collective participation in Korrika, although partially in the case of remembered well-being.

In line with the work of Wlodarczyk et al. (2021), the effect on social integration was facilitated by the indirect effect of QP through PES and enjoyment emotions. Emotions such as fun and joy are experienced and expressed collectively during collective gatherings, and it seems that they can also lead to a greater connection with other people.

The effect on social acceptance, social contribution and social actualization was facilitated by the indirect effect of QP through PES and kama muta. Kama muta can be considered a self-transcendent emotion directly oriented to horizontal relationships, relationships in which people feel like neighbors or similar in certain essential aspects of the Self (Zickfeld et al., 2019). In turn, it is likely that kama muta has also fueled the desire to take responsibility for the people and society they build (Fiske et al., 2017; Seibt et al., 2019; Landmann and Rohmann, 2020; Lizarazo Pereira et al., 2022), probably strengthening social contribution, that is, the feeling of being valuable and capable of improving society. Undoubtedly, kama muta emerges as a relevant emotion during collective gatherings whose further study seems valuable.

Regarding the effect on collective empowerment, this was facilitated by the indirect effect of QP through PES and self-transcendent emotions —wondered, grateful, inspired and hopeful—. Hope and inspiration, as well as awe or amazement at the vastness of social movements (in this case Basque) can be powerful emotions in fostering a sense of being able to achieve common goals and change the collective situation (Wlodarczyk et al., 2017b; Cohen-Chen and Van Zomeren, 2018; Landmann and Rohmann, 2020; Zumeta et al., 2021). These data support the idea that collective effervescence can be an important fuel for social movements, since one of the most important factors that explain collective action is collective efficacy.

The effect on recalled well-being was mainly facilitated by QP and not by PES or its proximal effects. However, the indirect total effect of PES and proximal effects was statistically significant, suggesting that the effect on remembered well-being is not only explained by mere collective participation, but also by collective effervescence. Indeed, PES partially mediated the relationship between QP and remembered well-being in a simple mediation.

It was expected that PES would show higher effects on the dependent variables. The lack of direct effects of PES on them may be explained because the brief scale only measures a small part of the original construct (Wlodarczyk et al., 2020). It’s possible, too, that the proximal effects overlap the effects of the PES because of the strong relationships between them, or that its effects act differently. Despite this, the proposed model has shown to be theoretically and empirically adequate.

Regarding the durability of the effect of participation, Durkheim underlined the need to repeat collective gatherings, since the effects dissipate over time. The few works that have analyzed this issue shows that the effects of participation in collective gatherings can last between 1 and 4 weeks (Páez et al., 2007, 2011, 2015; Rimé et al., 2010; Tewari et al., 2012; Khan et al., 2016), but even also up to 10 weeks (Bouchat et al., 2020). According to the data from our study, the effects of participation on social contribution, remembered well-being and collective empowerment dissipate at least 6–7 weeks after having participated. However, the effect of participation on social integration, social acceptance and social actualization is maintained (although it decreases). Thus, the participants of Korrika came back to their daily life with a greater feeling of belonging and being accepted by their community, with a better view of people, and with a greater trust in humanity and social progress. These are important indicators of the psychological health of people (Keyes, 1998; Postmes et al., 2019; Zabala et al., 2020; Páez and Oyanedel, 2021) that need to be taken into account.

Another interesting outcome that deserves to be highlighted from the results is the fact that while the effects of enjoyment emotions and kama muta on these variables (i.e., social integration, social acceptance and social actualization) dissipated before the 6–7 weeks after the participation, PES maintained its effect over this time. This suggests, that both enjoyment and self-transcendent emotions (kama muta included) have a powerful but fleeting effect. That is, they act tied to the moment where collective effervescence occurs (up to least 1 week according to the data of this study). Instead, the effect of PES can reach far beyond that instance, even up to 10 weeks according to the study of Bouchat et al. (2020). Therefore, it seems that the PES is what matters to maintain these positive psychological effects over time. However, further investigations are needed to confirm this relatively fleeting effect of emotions and the long-lasting effect of PES.

### 4.1. Limitations and future studies

The first limitation of this study is that, despite the fact that the model presented —PES as a central mechanism of collective processes— has shown to be both theoretically and statistically more adequate than other models (see Supplementary Table S3) and in line with both the theory and the results of previous studies, we cannot confirm a causality between the PES and the proximal and distal effects for methodological reasons. However, these results provide a basis to guide future studies in which this possible causal relationship is analyzed experimentally. As for the instruments used, it would probably have been more appropriate to use the full versions, as well as instruments that more fully measure the more specific aspects of personal well-being. In addition, it is likely that the instrument used to measure the PES did not measure the construct in its entirety (Wlodarczyk et al., 2020), and perhaps, for this reason, it has shown fewer effects than expected. Anyway, these instruments were selected with the aim of avoiding participant fatigue and the sample loss that commonly happen in longitudinal studies. Another limitation refers to the low reliability indices that social acceptance has shown, especially in T1, which in turn has had a negative influence on the fit indices of the social acceptance models. Therefore, this result should be viewed with caution. On the other hand, the relationships between the proximal effects of the PES and the dependent variables should also be considered with caution, as well as the possible explanations that have been presented, since these relationships could be different depending on the type of collective gathering analyzed. However, the results show a clear influence of collective effervescence on the effects of collective participation. Finally, in future studies, it would be fruitful to have a control group that had not participated in the meeting, since this would allow a better assessment of the results.

## 5. Conclusion

From the results of this study we can conclude, first, that participation in Korrika has positive psychological effects on social well-being and collective empowerment. In addition, the effects on social integration, social acceptance and social actualization can last for at least 6–7 weeks after participation. Second, we conclude that PES can be considered one of the central mechanisms of the effects of collective participation, and of special importance for their maintenance. Third, that kama muta emerges as a relevant emotion that should be paid attention to in future studies. And, finally, given the effect that collective gatherings (and their absence) have on people’s psychological health, we conclude that collective participation should be considered as another factor to be valued in health prevention programs.

## Data availability statement

The original contributions presented in the study are included in the article/Supplementary material, further inquiries can be directed to the corresponding author.

## Ethics statement

The studies involving human participants were reviewed and approved by University of the Basque Country’s Ethics Committee for Research involving Human Beings. The patients/participants provided their written informed consent to participate in this study.

## Author contributions

JZ, SC, and AP contributed to the design and implementation of the research. JZ collected the study’s data and analyzed and interpreted the data together with LZ and JP. JZ created the first draft of the manuscript which was then translated by IA-A. JZ, SC, AP, JP, LZ, and IA-A discussed the results, commented on the manuscript and contributed to the writing and editing of the final version of this manuscript. All authors contributed to the article and approved the submitted version.

## Funding

This research was carried out with funding provided by a Pre-doctoral Grant to JZ (PRE_2020_1_0338), by a Post-doc Grant from the UPV/EHU to LZ (DOCBERRI 20/23), by a grant from the Basque Government for Research Groups (Ref. IT-1598-22) and by a grant from the Spanish Ministry of Science and Innovation (Ref. PID2020-115738GB-I00).

## Conflict of interest

The authors declare that the research was conducted in the absence of any commercial or financial relationships that could be construed as a potential conflict of interest.

## Publisher’s note

All claims expressed in this article are solely those of the authors and do not necessarily represent those of their affiliated organizations, or those of the publisher, the editors and the reviewers. Any product that may be evaluated in this article, or claim that may be made by its manufacturer, is not guaranteed or endorsed by the publisher.

## Footnotes

^1^Korrika is organized by the Literacy and Euskaldunization Coordinator (AEK, in its acronym in Basque) and also seeks to raise funds for its network of Basque teaching centers (euskaltegiak). AEK is the most important organization in the field of euskaldunization and literacy of adults, and the only one that develops its activity throughout Basque Country, above administrative divisions. https://www.aek.eus/es-es/servicios/korrika

^2^Basque speakers whose mother tongue is Euskera account for 15.35% of the Basque Country’s population However, 29.62% of the inhabitants of the Basque Country have acquired language ability in Euskera, even though it is only practiced by 12.6% in public life (Sociolinguistic cluster, 2020).

^3^The first number between parentheses refers to the sample and the second number refers to the number of estimates (see Figures 1–6).

^4^Collinearity analysis showed adequate indices (tolerance >0.2, variance inflation factor < 4; Rovai et al., 2013). See Supplementary Table S5.

^5^Enjoyment emotions as mediator of the effect of PES on social integration in T3 controlling for pre-participation scores. Model fit: χ^2^ = (273, 49) = 111.382, *p* < 0.001, CFI = 0.964, TLI = 0.952, RMSEA = 0.068, SRMR = 0.040.

^6^Kama muta as mediator of the effect of PES on social acceptance in T3 controlling for pre-participation scores. Model fit: χ^2^ = (273, 130) = 376.737, *p* < 0.001, CFI = 0.910, TLI = 0.894, RMSEA = 0.083, SRMR = 0.060.

^7^Kama muta as mediator of the effect of PES on social actualization in T3 controlling for pre-participation scores. Model fit: χ^2^ = (273, 130) = 312.372, *p* < 0.001, CFI = 0.945, TLI = 0.935, RMSEA = 0.072, SRMR = 0.041.
