# Supplementary material for: Korrika, running in collective effervescence through the Basque Country: A model of collective processes and their positive psychological effects
Source: Front Psychol. 2023 Feb 10;14:1095763. doi: 10.3389/fpsyg.2023.1095763 (PMC9950557; doi:10.3389/fpsyg.2023.1095763)
Supplement: Supplementary file 1 [file Table_1.DOCX]

Supplementary Material

| Supplementary Table S1  *Means comparison in sex, age and the dependent variables in T1 between participants who answered the three questionaries and participants who answered T1 and T2 questionaries.* | | | | |  |
| --- | --- | --- | --- | --- | --- |
| Variables | T1-T2-T3  (N = 276)  *M* (*SD*) | T1-T2  (N = 129)  *M* (*SD*) | *F*(2, 403) | *p* |  |
| Sex | 1.68 (0.49) | 1.70 (0.49) | 0.129 | .709 |  |
| Age | 39.42 (11.66) | 39.50 (11.94) | 0.001 | .397 |  |
| Social integration | 3.97 (0.61) | 3.96 (0.57) | 0.074 | .870 |  |
| Social acceptance | 3.22 (0.52) | 3.15 (0.62) | 1.533 | .264 |  |
| Social contribution | 3.64 (0.65) | 3.51 (0.69) | 1.822 | .074 |  |
| Social actualization | 2.55 (0.77) | 2.44 (0.75) | 0.079 | .161 |  |
| Remembered well-being | 7.89 (1.13) | 7.97 (1.04) | 0.327 | .501 |  |
| Collective empowerment | 7.80 (1.25) | 7.76 (1.32) | .001 | .725 |  |

As we can see, there is no difference between these two groups. So, we consider it appropriate do the structural equation modeling with all the participants (N = 405) that answered the T1 and T2 questionaries.

| Supplementary Table S2  *Means, standard deviations, and correlations between mechanisms during the participation (T2) and T1 dependent variables* | | | | | | | | | | | | | |
| --- | --- | --- | --- | --- | --- | --- | --- | --- | --- | --- | --- | --- | --- |
| Variable | *M* | *SD* | 1 | 2 | 3 | 4 | 5 | 6 | 7 | 8 | 9 | 10 |  |
|  |  |  |  |  |  |  |  |  |  |  |  |  |  |
| 1. QP^T2^ | 6.19 | 0.92 |  |  |  |  |  |  |  |  |  |  |  |
|  |  |  |  |  |  |  |  |  |  |  |  |  |  |
| 2. PES^T2^ | 5.92 | 1.04 | .52** |  |  |  |  |  |  |  |  |  |  |
|  |  |  | [.44, .59] |  |  |  |  |  |  |  |  |  |  |
| 3. KM^T2^ | 4.93 | 1.38 | .44** | .67** |  |  |  |  |  |  |  |  |  |
|  |  |  | [.36, .52] | [.61, .72] |  |  |  |  |  |  |  |  |  |
| 4. STE^T2^ | 4.17 | 0.66 | .58** | .69** | .62** |  |  |  |  |  |  |  |  |
|  |  |  | [.51, .64] | [.64, .74] | [.56, .68] |  |  |  |  |  |  |  |  |
| 5. EE^T2^ | 4.47 | 0.62 | .57** | .60** | .52** | .70** |  |  |  |  |  |  |  |
|  |  |  | [.50, .63] | [.53, .66] | [.45, .59] | [.65, .75] |  |  |  |  |  |  |  |
| 6. S. I^T1^ | 3.98 | 0.60 | .17** | .29** | .22** | .21** | .18** |  |  |  |  |  |  |
|  |  |  | [.08, .27] | [.19, .37] | [.13, .31] | [.11, .30] | [.08, .27] |  |  |  |  |  |  |
| 7. S. AC^T1^ | 3.20 | 0.60 | .04 | .10* | .04 | .08 | .04 | .37** |  |  |  |  |  |
|  |  |  | [-.06, .13] | [.01, .20] | [-.06, .14] | [-.02, .18] | [-.06, .14] | [.28, .45] |  |  |  |  |  |
| 8. S. C^T1^ | 3.60 | 0.67 | .05 | .19** | .24** | .15** | .07 | .45** | .19** |  |  |  |  |
|  |  |  | [-.05, .15] | [.09, .28] | [.15, .33] | [.05, .24] | [-.03, .17] | [.37, .53] | [.10, .28] |  |  |  |  |
| 9. S. ACT^T1^ | 2.52 | 0.77 | .00 | .08 | .06 | .16** | .07 | .24** | .40** | .13** |  |  |  |
|  |  |  | [-.10, .10] | [-.02, .17] | [-.03, .16] | [.06, .25] | [-.03, .17] | [.15, .33] | [.31, .48] | [.03, .22] |  |  |  |
| 10. RWB^T1^ | 7.89 | 1.11 | .16** | .23** | .12* | .19** | .19** | .37** | .30** | .30** | .26** |  |  |
|  |  |  | [.06, .25] | [.13, .32] | [.02, .22] | [.09, .28] | [.09, .28] | [.28, .45] | [.21, .39] | [.21, .39] | [.16, .35] |  |  |
| 11. CE^T1^ | 7.80 | 1.28 | .12* | .22** | .16** | .23** | .21** | .33** | .33** | .20** | .34** | .50** |  |
|  |  |  | [.02, .21] | [.13, .31] | [.06, .25] | [.14, .32] | [.11, .30] | [.24, .42] | [.24, .41] | [.10, .29] | [.25, .42] | [.43, .57] |  |
| *Note.* *M* and *SD* are used to represent mean and standard deviation, respectively. Values in square brackets indicate the 95% confidence interval for each correlation. QP; Quality of participation, PES; Perceived emotional synchrony, KM; Kama muta, STE; Self-transcendent emotions, EE; Enjoyment emotions, S. I; Social integration, S. AC; Social acceptance, S. C; Social contribution, S. ACT; Social actualization, CE; Collective empowerment. * indicates *p* < .05. ** indicates *p* < .01. | | | | | | | | | | | | | |

| Supplementary Table S3  *Means, standard deviations, and correlations during the participation in Korrika (T2)* | | | | | | | | | | | | | |
| --- | --- | --- | --- | --- | --- | --- | --- | --- | --- | --- | --- | --- | --- |
| Variables | *M* | *SD* | 1 | 2 | 3 | 4 | 5 | 6 | 7 | 8 | 9 | 10 |  |
|  |  |  |  |  |  |  |  |  |  |  |  |  |  |
| 1. QP^T2^ | 6.19 | 0.92 |  |  |  |  |  |  |  |  |  |  |  |
|  |  |  |  |  |  |  |  |  |  |  |  |  |  |
| 2. PES^T2^ | 5.92 | 1.04 | .52** |  |  |  |  |  |  |  |  |  |  |
|  |  |  | [.44, .59] |  |  |  |  |  |  |  |  |  |  |
| 3. KM^T2^ | 4.93 | 1.38 | .44** | .67** |  |  |  |  |  |  |  |  |  |
|  |  |  | [.36, .52] | [.61, .72] |  |  |  |  |  |  |  |  |  |
| 4. STE^T2^ | 4.17 | 0.66 | .58** | .69** | .62** |  |  |  |  |  |  |  |  |
|  |  |  | [.51, .64] | [.64, .74] | [.56, .68] |  |  |  |  |  |  |  |  |
| 5. EE^T2^ | 4.47 | 0.62 | .57** | .60** | .52** | .70** |  |  |  |  |  |  |  |
|  |  |  | [.50, .63] | [.53, .66] | [.45, .59] | [.65, .75] |  |  |  |  |  |  |  |
| 6. S. I^T2^ | 4.14 | 0.65 | .27** | .42** | .37** | .35** | .36** |  |  |  |  |  |  |
|  |  |  | [.18, .36] | [.33, .50] | [.28, .45] | [.26, .44] | [.28, .44] |  |  |  |  |  |  |
| 7. S. AC^T2^ | 3.41 | 0.67 | .18** | .26** | .29** | .25** | .20** | .42** |  |  |  |  |  |
|  |  |  | [.09, .28] | [.17, .35] | [.20, .37] | [.16, .34] | [.10, .29] | [.34, .50] |  |  |  |  |  |
| 8. S. C^T2^ | 3.74 | 0.73 | .22** | .31** | .39** | .31** | .20** | .52** | .42** |  |  |  |  |
|  |  |  | [.13, .31] | [.22, .40] | [.30, .47] | [.22, .39] | [.11, .30] | [.45, .59] | [.33, .49] |  |  |  |  |
| 9. S. ACT^T2^ | 2.94 | 0.83 | .16** | .27** | .31** | .30** | .24** | .40** | .61** | .42** |  |  |  |
|  |  |  | [.06, .25] | [.17, .35] | [.22, .39] | [.21, .39] | [.15, .33] | [.32, .48] | [.54, .67] | [.33, .50] |  |  |  |
| 10. RWB^T2^ | 7.97 | 1.16 | .19** | .30** | .20** | .25** | .23** | .42** | .29** | .37** | .29** |  |  |
|  |  |  | [.10, .29] | [.21, .39] | [.11, .30] | [.16, .34] | [.13, .32] | [.33, .49] | [.20, .38] | [.28, .45] | [.19, .37] |  |  |
| 11. CE^T2^ | 8.04 | 1.34 | .21** | .34** | .33** | .37** | .32** | .43** | .33** | .36** | .39** | .48** |  |
|  |  |  | [.11, .30] | [.26, .43] | [.24, .41] | [.29, .46] | [.23, .40] | [.34, .50] | [.24, .42] | [.28, .45] | [.30, .47] | [.40, .55] |  |
| *Note.* *M* and *SD* are used to represent mean and standard deviation, respectively. Values in square brackets indicate the 95% confidence interval for each correlation. QP; Quality of participation, PES; Perceived emotional synchrony, KM; Kama muta, STE; Self-transcendent emotions, EE; Enjoyment emotions, S. I; Social integration, S. AC; Social acceptance, S. C; Social contribution, S. ACT; Social actualization, CE; Collective empowerment * indicates *p* < .05. ** indicates *p* < .01. | | | | | | | | | | | | | |

| Supplementary Table S4  *Means, standard deviations, and correlations between mechanisms during the participation (T2) and T3 dependent variables* | | | | | | | | | | | | | |
| --- | --- | --- | --- | --- | --- | --- | --- | --- | --- | --- | --- | --- | --- |
| Variable | *M* | *SD* | 1 | 2 | 3 | 4 | 5 | 6 | 7 | 8 | 9 | 10 |  |
|  |  |  |  |  |  |  |  |  |  |  |  |  |  |
| 1. QP^T2^ | 6.19 | 0.92 |  |  |  |  |  |  |  |  |  |  |  |
|  |  |  |  |  |  |  |  |  |  |  |  |  |  |
| 2. PES^T2^ | 5.92 | 1.04 | .52** |  |  |  |  |  |  |  |  |  |  |
|  |  |  | [.44, .59] |  |  |  |  |  |  |  |  |  |  |
| 3. KM^T2^ | 4.93 | 1.38 | .44** | .67** |  |  |  |  |  |  |  |  |  |
|  |  |  | [.36, .52] | [.61, .72] |  |  |  |  |  |  |  |  |  |
| 4. STE^T2^ | 4.17 | 0.66 | .58** | .69** | .62** |  |  |  |  |  |  |  |  |
|  |  |  | [.51, .64] | [.64, .74] | [.56, .68] |  |  |  |  |  |  |  |  |
| 5. EE^T2^ | 4.47 | 0.62 | .57** | .60** | .52** | .70** |  |  |  |  |  |  |  |
|  |  |  | [.50, .63] | [.53, .66] | [.45, .59] | [.65, .75] |  |  |  |  |  |  |  |
| 6. S. I^T3^ | 4.06 | 0.66 | .09 | .25** | .14* | .19** | .15* |  |  |  |  |  |  |
|  |  |  | [-.03, .20] | [.14, .36] | [.02, .25] | [.08, .30] | [.03, .27] |  |  |  |  |  |  |
| 7. S. ACC^T3^ | 3.32 | 0.57 | .01 | .20** | .13* | .15* | .06 | .47** |  |  |  |  |  |
|  |  |  | [-.11, .13] | [.08, .31] | [.01, .24] | [.03, .26] | [-.06, .17] | [.37, .56] |  |  |  |  |  |
| 8. S. C^T3^ | 3.69 | 0.68 | .08 | .20** | .14* | .16** | .07 | .51** | .44** |  |  |  |  |
|  |  |  | [-.04, .19] | [.09, .32] | [.02, .25] | [.04, .27] | [-.05, .19] | [.41, .59] | [.34, .53] |  |  |  |  |
| 9. S. ACT^T3^ | 2.90 | 0.78 | .01 | .11 | .02 | .16** | .08 | .19** | .47** | .33** |  |  |  |
|  |  |  | [-.11, .13] | [-.01, .23] | [-.10, .14] | [.04, .27] | [-.04, .20] | [.07, .30] | [.37, .56] | [.22, .43] |  |  |  |
| 10. RWB^T3^ | 7.83 | 1.15 | .20** | .27** | .11 | .24** | .23** | .52** | .44** | .48** | .32** |  |  |
|  |  |  | [.09, .31] | [.16, .38] | [-.01, .23] | [.13, .35] | [.12, .34] | [.43, .60] | [.34, .53] | [.39, .57] | [.21, .43] |  |  |
| 11. CE^T3^ | 7.81 | 1.26 | .17** | .25** | .16** | .33** | .28** | .41** | .41** | .38** | .32** | .50** |  |
|  |  |  | [.05, .28] | [.13, .35] | [.04, .27] | [.22, .43] | [.17, .39] | [.31, .50] | [.31, .50] | [.27, .47] | [.21, .42] | [.40, .58] |  |
| *Note.* *M* and *SD* are used to represent mean and standard deviation, respectively. Values in square brackets indicate the 95% confidence interval for each correlation. QP; Quality of participation, PES; Perceived emotional synchrony, KM; Kama muta, STE; Self-transcendent emotions, EE; Enjoyment emotions, S. I; Social integration, S. AC; Social acceptance, S. C; Social contribution, S. ACT; Social actualization, CE; Collective empowerment * indicates *p* < .05. ** indicates *p* < .01. | | | | | | | | | | | | | |

| Supplementary Table S5.  *Collinearity Statistics: Tolerance Test and Variance Inflation Factor (VIF)* | | |
| --- | --- | --- |
| *Variables* | *Tolerance* | *VIF* |
| Quality of participation | .601 | 1.663 |
| Perceived emotional synchrony | .408 | 2.448 |
| Kama muta | .504 | 1.986 |
| Self-transcendent emotions | .364 | 2.746 |
| Enjoyment emotions | .455 | 2.196 |
| Social integration^T1^ | .921 | 1.086 |
| *Note*: Dependent variable: Social Integration^T2^ | | |

**Supplementary Figure 1**

*Enjoyment emotions as mediator of the effect of PES on social integration in T3 controlling for pre-participation scores.*

*
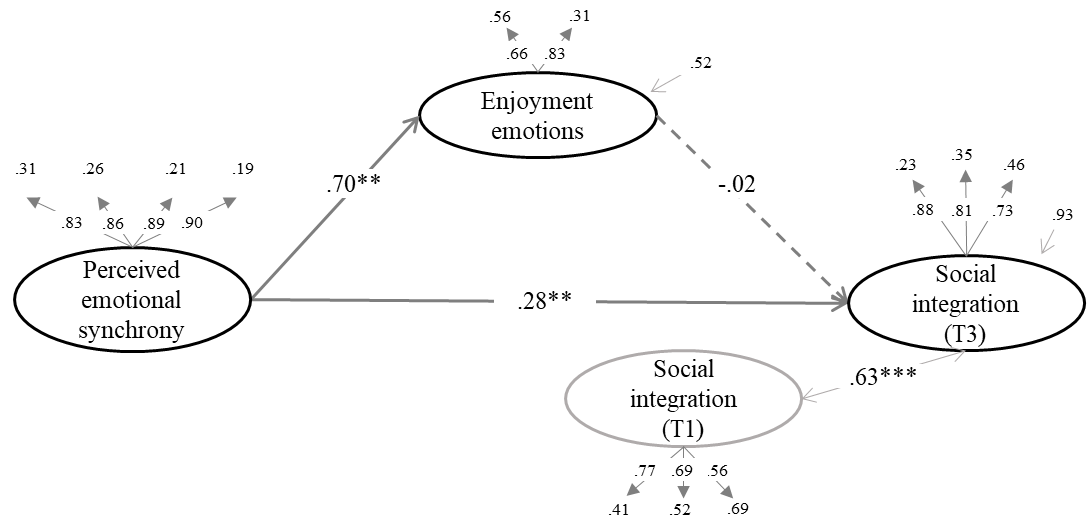
Note*. Model fit: χ2 = (273, 49) = 111.382, *p* < 0.001, CFI = 0.964, TLI = 0.952, RMSEA = 0.068, SRMR= 0.040.

**Supplementary Figure 2**

*Kama muta as mediator of the effect of PES on social acceptance in T3 controlling for pre-participation scores.*

*
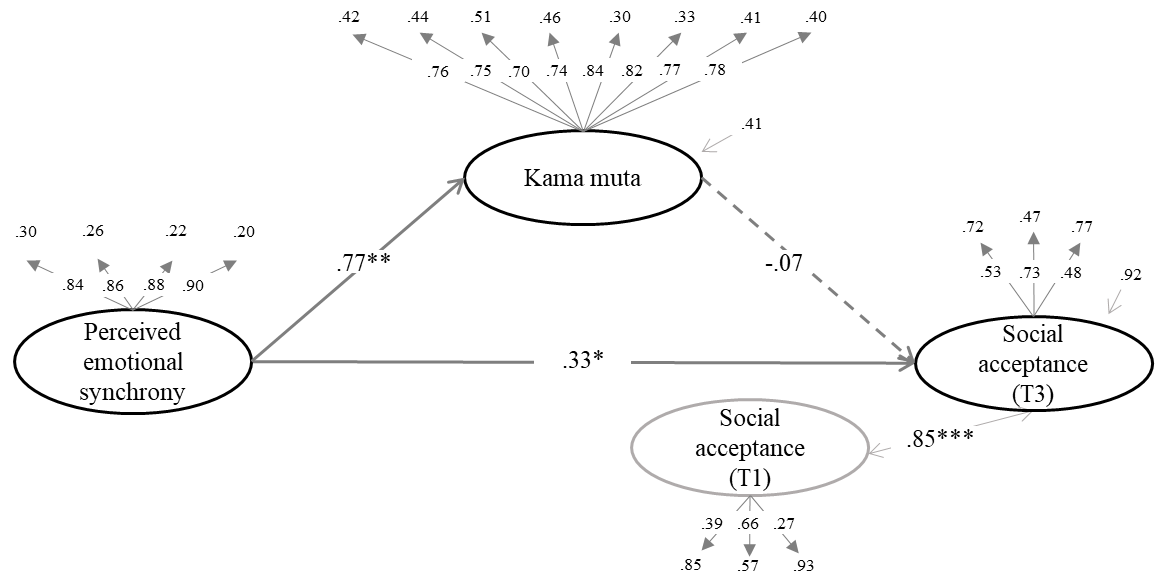
*

*Note*. Model fit: χ2 = (273, 130) = 376.737, *p* < 0.001, CFI = 0.910, TLI = 0.894, RMSEA = 0.083, SRMR= 0.060.

**Supplementary Figure 3**

*Kama muta as mediator of the effect of PES on social actualization in T3 controlling for pre-participation scores.*

*
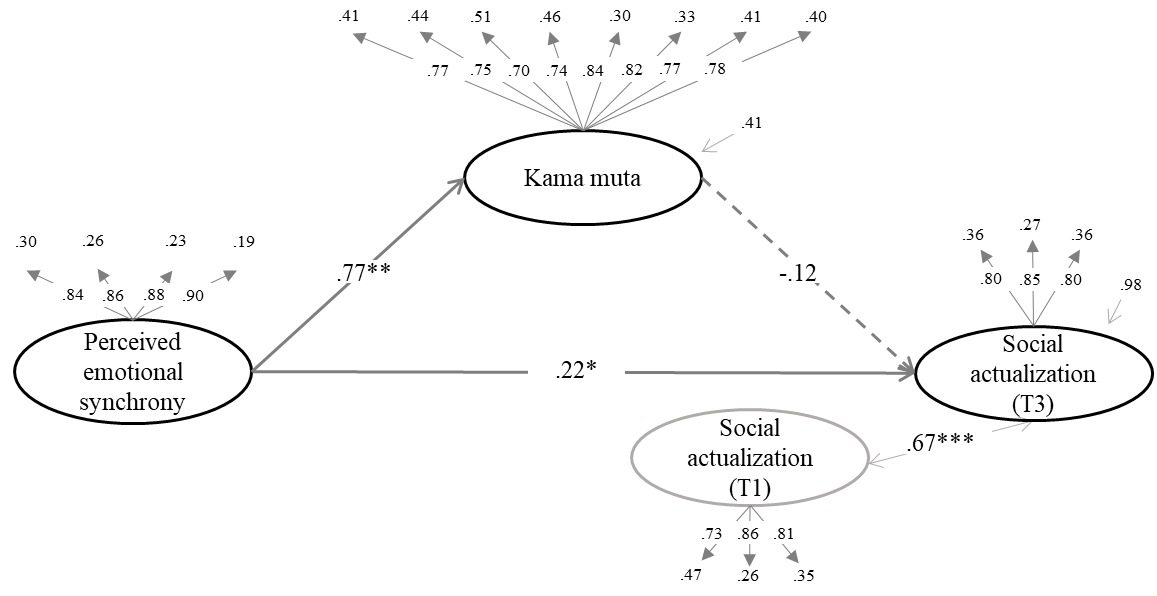
*

*Note*. Model fit: χ2 = (273, 130) = 312.372, *p* < 0.001, CFI = 0.945, TLI = 0.935, RMSEA = 0.072, SRMR= 0.041.

**
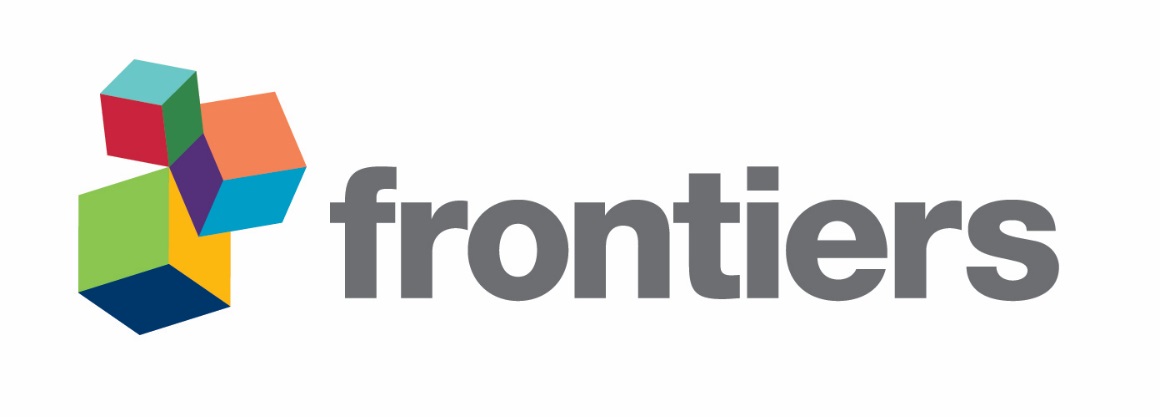
**
